# Genetic Association Study of Adiposity and Melanocortin-4 Receptor (*MC4R*) Common Variants: Replication and Functional Characterization of Non-Coding Regions

**DOI:** 10.1371/journal.pone.0096805

**Published:** 2014-05-12

**Authors:** Daniel S. Evans, Melissa A. Calton, Mee J. Kim, Pui-Yan Kwok, Iva Miljkovic, Tamara Harris, Annemarie Koster, Yongmei Liu, Gregory J. Tranah, Nadav Ahituv, Wen-Chi Hsueh, Christian Vaisse

**Affiliations:** 1 California Pacific Medical Center Research Institute, San Francisco, California, United States of America; 2 Diabetes Center and Department of Medicine, University of California, San Francisco, California, United States of America; 3 Department of Bioengineering and Therapeutic Sciences and Institute for Human Genetics, University of California, San Francisco, California, United States of America; 4 Cardiovascular Research Institute, Institute for Human Genetics, and Department of Dermatology, University of California, San Francisco, California, United States of America; 5 Department of Epidemiology, University of Pittsburgh, Pittsburgh, Pennsylvania, United States of America; 6 National Institute on Aging, Bethesda, Maryland, United States of America; 7 Department of Epidemiology and Prevention, Division of Public Health Sciences, Wake Forest University, Winston-Salem, North Carolina, United States of America; University of Catanzaro Magna Graecia, Italy

## Abstract

Common genetic variants 3′ of *MC4R* within two large linkage disequilibrium (LD) blocks spanning 288 kb have been associated with common and rare forms of obesity. This large association region has not been refined and the relevant DNA segments within the association region have not been identified. In this study, we investigated whether common variants in the *MC4R* gene region were associated with adiposity-related traits in a biracial population-based study. Single nucleotide polymorphisms (SNPs) in the *MC4R* region were genotyped with a custom array and a genome-wide array and associations between SNPs and five adiposity-related traits were determined using race-stratified linear regression. Previously reported associations between lower BMI and the minor alleles of rs2229616/Val103Ile and rs52820871/Ile251Leu were replicated in white female participants. Among white participants, rs11152221 in a proximal 3′ LD block (closer to *MC4R*) was significantly associated with multiple adiposity traits, but SNPs in a distal 3′ LD block (farther from *MC4R*) were not. In a case-control study of severe obesity, rs11152221 was significantly associated. The association results directed our follow-up studies to the proximal LD block downstream of *MC4R*. By considering nucleotide conservation, the significance of association, and proximity to the *MC4R* gene, we identified a candidate *MC4R* regulatory region. This candidate region was sequenced in 20 individuals from a study of severe obesity in an attempt to identify additional variants, and the candidate region was tested for enhancer activity using *in vivo* enhancer assays in zebrafish and mice. Novel variants were not identified by sequencing and the candidate region did not drive reporter gene expression in zebrafish or mice. The identification of a putative insulator in this region could help to explain the challenges faced in this study and others to link SNPs associated with adiposity to altered *MC4R* expression.

## Introduction

Obesity has been increasing in prevalence worldwide and is a risk factor for many poor health outcomes [1], [2]. Obesity results from the interaction between genetic and non-genetic factors. Studies of severe and common forms of obesity have demonstrated that the Melanocortin-4 Receptor (*MC4R*) is an important regulator of obesity and adiposity [3]. *MC4R* belongs to a family of seven trans-membrane G-protein-coupled receptors (GPCR) and is expressed at low levels in hypothalamic nuclei involved in the regulation of food intake [4]. *MC4R* regulates food intake by integrating a satiety signal provided by its agonist α-MSH and an orexigenic signal provided by its antagonist Agouti-related protein (AGRP) [5], [6]. These ligands are expressed in distinct neuronal populations of the arcuate nucleus of the hypothalamus and are regulated by the adipocyte-secreted hormone, leptin, to control food intake and maintain long-term energy homeostasis [7]. Mice lacking both alleles of *mc4r* (*mc4r* −/− mice) develop a maturity onset hyperphagic obesity syndrome by 10 weeks of age, while mice heterozygous for a *mc4r* deletion (*mc4r* +/− mice) show an intermediate obese phenotype [6].

Genetic variants within the *MC4R* coding region have been found to be associated with severe and common forms of obesity [3]. Rare mutations in the *MC4R* coding region account for a significant number of severe obesity cases [3], [8], [9], [10]. More common, but still quite rare (minor allele frequency (MAF) <5% in most populations) *MC4R* non-synonymous SNPs (nsSNPs) (rs2229616/Val103Ile and rs52820871/Ile251Leu) have been reproducibly associated with a protective effect from severe and common forms of obesity [11], [12], [13], [14], [15], [16]. Functional studies indicate that the 251Leu allele increases MC4R basal activity and the 103Ile allele decreases MC4R antagonist potency while also increasing MC4R agonist potency [17]. These biochemical effects result in elevated MC4R function, which is consistent with the association between these variants and a lower body weight.

In addition to variants within the *MC4R* coding region, common variants outside of the coding region have been associated with common and severe forms of obesity. Meta-analyses of genome-wide association studies (GWAS) conducted in Caucasians have identified common variants in two large linkage disequilibrium (LD) blocks 3′ of the *MC4R* coding region that are associated with adiposity and anthropometric traits [3], [18], [19], [20], [21], [22], [23]. The most significant association signal in the proximal 3′ LD block (closer to *MC4R*) is rs17700633, and in the distal 3′ LD block (farther from *MC4R*) is rs17782313 [19]. Multiple SNPs in high LD with rs17782313 (rs17700144, HapMap Phase 3 CEU *r* ^2^ = 0.83; rs12970134, HapMap Phase 3 CEU *r* ^2^ = 0.84) have also been associated with adiposity-related traits [18], [22], [23]. In addition to common forms of obesity, rs17782313 and rs17700144 have also been associated with early-onset severe obesity [16], [24].

While recent GWAS efforts in populations of European descent have been very successful at identifying the 288 kb association region that encompasses both LD blocks located 21 kb 3′ of *MC4R*, there has been little success at refining this association region or assigning a functional role to non-coding variants in these regions. Conditional analysis indicates that at least a small degree of dependence might exist between SNPs in the proximal and distal LD blocks, even though LD would suggest otherwise [19]. It has been argued that synthetic associations with *MC4R* nsSNPs are not likely to underlie the associations between SNPs 3′ of *MC4R* and obesity [25]. Thus, the identity of causal variants that might underlie common SNP associations in the *MC4R* non-coding region remains unknown.

Refining the large association region 3′ of *MC4R* and evaluating the biological role of DNA in this region could aid in the identification of causal risk alleles near *MC4R*. To this end, we investigated the association of common SNPs within and surrounding the *MC4R* gene with multiple adiposity-related traits in the Health ABC study, a biracial population-based cohort. SNP associations with severe obesity in an independent study were also examined. By considering nucleotide conservation, the significance of association, and proximity to the *MC4R* gene, we identified a candidate *MC4R* regulatory region. This candidate region was sequenced in 20 individuals from a study of severe obesity in an attempt to identify additional variants, and the candidate region was tested for enhancer activity using *in vivo* enhancer assays in zebrafish and mice. Data from the ENCODE project [26] were used to gain further insight into the biological function of this DNA region.

## Materials and Methods

### Ethics Statement

The Health ABC study protocol was approved by the institutional review boards at the University of Pittsburgh and the University of Tennessee, Memphis, and written informed consent was obtained from all participants. The severe obesity study protocol was approved by the UCSF Committee on Human Research, and written informed consent was obtained from all participants. All animal work protocols were approved by the UCSF Institutional Animal Care and Use Committee (Approval Number: AN100466-01A).

### Study populations

The Health, Aging, and Body Composition (Health ABC) study is a population-based prospective study of 3,075 men and women (48.5% male; 41.7% African American) aged 70 to 79 years at baseline, residing in Pittsburgh, PA and Memphis, TN. All participants were well-functioning at the time of entry into the study; they reported no difficulty walking a quarter of a mile or walking up 10 steps without resting. Data used in the present study were obtained from the baseline examination, during 1997–1998. Adiposity-related measures in these participants have been described previously [27]. Briefly, percentage of total body fat was assessed by DXA and abdominal visceral fat area (visceral fat) and abdominal subcutaneous fat area (subcutaneous fat) (cm^2^) were assessed using the computed tomography scan image measured at the L4-L5 disk space. Serum leptin was measured by radioimmunoassay (Linco Research Inc, St Charles, MO) in the morning from participants who fasted overnight.

Severely obese participants were selected from an ongoing UCSF study, as previously described [28].

### TagSNP selection

In order to capture the genetic variation in the *MC4R* coding region and flanking non-coding DNA, SNPs were selected using HapMap Phase 2 (release 20) project data (www.hapmap.org/). By considering conservation between human and mouse genomes, non-coding regions up to 32.5 kb from the 5′ end (base pair 56,223,516, Reference assembly, NCBI genome build 36.3) and 21.3 kb from the 3′ end (base pair 56,168,229) of *MC4R* were used to select tagSNPs, using the program Tagger [29]. TagSNPs were chosen based on having known or predicted alterations of gene or protein function and having strong LD (r^2^≥0.8) with other SNPs. In order to select tagSNPs appropriate for use with a biracial cohort such as the Health ABC Study, tagSNPs were first selected using CEU (Caucasian/European) SNP genotypes, and these tagSNPs were then added to the tagSNP selection using YRI (Yoruban) SNP genotypes. Thirty-seven SNPs in or near *MC4R* were selected for genotyping using the Illumina Golden Gate Assay.

### Genotyping and quality control

All tagSNPs except for rs17782313 were genotyped using the Illumina Golden Gate Assay (Illumina, San Diego, CA, USA) from DNA isolated from participants of the Health ABC Study and the UCSF obesity study. Rs17782313 was genotyped with a Taqman assay from ABI using a stock kit. All samples that produced a genotype for rs17782313 were used to analyze that SNP. For the Illumina Golden Gate Assay, samples with a missing call rate greater than 10% were excluded. Five percent of the DNA samples were genotyped in duplicate to estimate genotyping error rate, and SNPs with more than one discrepancy between duplicate samples (0.7% error rate) were excluded from analysis; none of the SNPs were excluded based on this criterion. SNPs were also removed if the HWE *P*-value in white participants was <0.001 (Bonferroni corrected *P*-value of 0.05 corrected for 38 SNPs); none of the SNPs were excluded based on this criterion. 13 SNPs in Health ABC white participants and 5 SNPs in Health ABC black participants were excluded from analysis based on MAF <0.05.

To extend our SNP coverage into both LD blocks located 3′ to *MC4R*, we examined genome-wide SNP genotypes that were previously assayed in Health ABC participants using the Illumina Human 1M-Duo array. Genotypes were called using Illumina BeadStudio. Samples were excluded from the dataset for the reasons of sample failure, genotypic sex mismatch, and first-degree relative of an included individual based on genotype data. SNPs were excluded if the call rate was <97%, HWE *P*-value <10^−6^, or MAF <0.01.

Individual level genetic data from the genome-wide SNP dataset from Health ABC is available through controlled access from dbGaP (dbGaP Study accession phs000169.v1.p1). Individual level Health ABC genetic data from the *MC4R* candidate gene SNP genotype data is available through Health ABC's coordinating center website (http://www.keeptrack.ucsf.edu/).

### Sequencing putative MC4R regulatory region

Among the severely obese patients from the UCSF study that were homozygous for the rs11152221 C allele (major allele) or the T allele (minor allele), ten CC homozygotes and ten TT homozygotes were randomly selected for sequencing using the R function sample, which employs the Mersenne-Twister pseudorandom number generator. The forward primer 5′-GGCTGCTGCTGGGGTCAACA-3′and reverse primer 5′-ACCCACCATCCCATCTGTGCGA-3′ were used in PCR to amplify the 1.25 kb region of interest (NCBI build 36: chromosome 18: 56,168,229–56,169,479). The sequencing reaction was performed with the BigDye terminator kit (Applied Biosystems, Foster City, CA) under the standard manufacturer's conditions. Sequencing was performed on an ABIPRISM 3700 automated DNA sequencer (Applied Biosystems). Sanger sequencing data can be fully reconstructed from the description in the results.

### Cloning, transgenics, and enhancer assays

These studies were carried out in strict accordance with the recommendations in the Guide for the Care and Use of Laboratory Animals of the National Institutes of Health and all efforts were made to minimize suffering. The protocols were approved by the UCSF Institutional Animal Care and Use Committee (Approval Number: AN100466-01A).

We PCR amplified the same DNA region that was sequenced (NCBI build 36: chromosome 18: 56,168,229–56,169,479) using the forward primer 5′-AACTCGAGGGCTGCTGCTGGGGTCAACA-3′ and reverse primer: 5′-GGCTCGAGACCCACCATCCCATCTGTGCGA-3′ from genomic DNA of a patient from the UCSF study who was homozygous for the rs11152221 C allele. The sequence was sequence verified for having the proper allele. For zebrafish transgenics, the PCR product was cut with XhoI and ligated into the E1B-GFP-Tol2 enhancer assay vector [30]. The plasmid DNA was cleaned for endotoxins using the Qiagen EndoFree Plasmid Midi kit.

Zebrafish injections were performed using standard procedures as previously described [31]. The injection mix contained 1 uL 125 ng/uL endotoxin-free plasmid DNA, 1 uL 175 ng/uL Tol2 RNA, 2 uL sterile water, and 1 uL 2% Phenol red. Embryo injections were performed four independent times and at least 50 embryos were injected each time. Zebrafish were examined at 24 hrs, 48 hrs, and 72 hrs post-fertilization for GFP expression, and at least 85 healthy surviving embryos were analyzed at each time point. Imaging of zebrafish was done using a Lumar V12 Stereomicroscope (Carl Zeiss) with Axio Vision Rel. 4.4 (Carl Zeiss).

For mouse transgenics, the candidate enhancer region was PCR amplified from human genomic DNA, as described above, and digested with XhoI and SmaI. The region was cloned into the Hsp68-promoter-LacZ reporter vector [32]. Transgenic mouse embryos were generated through Cyagen Biosciences, Inc. using standard procedures [33] and embryos at day 15 were stained for LacZ expression as in [34]. The embryos were then processed and imbedded in paraffin, sectioned (7 um thickness) and counterstained with neutral fast red for visualization by light microscopy (Carl Zeiss) with Axio Vision Rel. 4.4.

### Statistical Analysis

For the BMI, percent body fat, and leptin outcomes, the effects of age, sex, recruitment site, prevalent diabetes status, weekly levels of calculated physical activity, smoking and drinking habits, and education levels were adjusted for in the regression analysis. Leptin, visceral fat and subcutaneous fat were transformed by taking the square-root to produce normal distributions. To identify associations with leptin independent of percent body fat, leptin was subsequently analyzed adjusting for the percent body fat. To adjust for overall body size, baseline height and weight were included as covariates for abdominal visceral and subcutaneous fat.

For association analysis using tagSNPs from the Illumina Golden Gate assay, the appropriate mode of inheritance was determined by examining parameter estimates from a genotypic 2df test. All SNPs are modeled with an additive mode of inheritance, except for rs11152221, which was modeled as dominant, and rs1943225, which was modeled as recessive. To avoid population stratification, all analyses were performed in whites and blacks separately. The first two principal components determined from principal component analysis (PCA) using genome-wide SNP data did not impact tagSNP association effect estimates. *P*-values less than 0.05 were deemed significant for sex-interactions and associations between replication SNPs (rs52820871, rs2229616, and rs17782313) and adiposity-related traits. Significance of associations using tagSNPs was corrected for multiple hypothesis testing by obtaining empirical *P*-values through permutation testing by the min*P* procedure using 100,000 replicates [35]. Logistic regression models that adjusted for the effects of age, sex, recruitment site, prevalent diabetes status, weekly levels of calculated physical activity, smoking and drinking habits, and education levels were used to determine the association between rs11152221 and obesity using cases (BMI≥30) and controls (BMI<30) identified from participants in the Health ABC study.

Association between rs11152221 and obesity was also examined using cases (BMI≥30) from self-identified Caucasian subjects from the UCSF study of severe obesity and controls (BMI<30) from white participants of the Health ABC study. Logistic regression models included sex as a covariate.

For the SNP association analysis of the extended *MC4R* region, directly genotyped and imputed SNPs on chromosome 18 from position 55,850,000–56,230,000 (NCBI build 36) were selected from genome-wide genotyped and imputed SNP data that were previously obtained in Health ABC participants. Genotype imputation was performed using MACH (v. 1.0.16) with the HapMap CEU Phase 2 release 22 build 36 haplotypes in Health ABC white participants and a 1∶1 mixture of HapMap CEU:YRI Phase 2 release 22 build 36 haplotypes in Health ABC black participants. Imputed SNPs with an MAF<0.05 or an observed:expected variance ratio <0.3 were removed. To adjust the significance threshold for multiple testing of 304 genotyped and imputed SNPs in the *MC4R* region, a Bonferroni correction was applied using the number of independent SNPs, which was determined using Tagger to select tagSNPs in the region of interest from HapMap phase 2 release 24 CEU and YRI genotypes (SNPs with MAF<0.05 excluded, pair-wise tagging with an r^2^ threshold of 0.8) [29]. There were 55 tagSNPs for CEU genotypes and 119 tagSNPs for YRI genotypes, resulting in significance thresholds of 9×10^−4^ and 4×10^−4^ in Health ABC white and black participants, respectively. Selection of tagSNPs using HapMap phase 3 ASW (individuals of African ancestry in Southwest USA) genotypes yielded 97 tagSNPs, but in an effort to be conservative, the significance threshold based on YRI genotypes was adopted for Health ABC black participants. The first two principal components determined from PCA of genome-wide SNP data were included in regression models. Covariates used were the same as in the analysis of tagSNPs from the Illumina Golden Gate assay. LD of imputed allele dosages was visualized by constructing a correlation matrix (Pearson's *r^2^*) of the imputed allele dosage data for SNPs in the region of interest that passed QC, then plotting the correlation matrix as a heatmap using the LDheatmap R package. Nucleotide conservation between human and mouse genomes was obtained using the VISTA browser [36]. All regression analyses were performed using R software (www.r-project.org).

Publicly available expression quantitative trait loci (eQTL) data from CEU and YRI HapMap3 lymphoblastoid cell lines were accessed using Genevar [37], [38]. On chromosome 18, position 55,850,000–56,230,000 (NCBI build 36), SNP associations with *MC4R* expression were determined using Spearman's rank correlation coefficient and association significance was assessed using a *t*-statistic and a *t*-distribution with n-2 degrees of freedom. The eQTL significance level was adjusted for multiple testing using the Bonferroni correction by dividing 0.05 by the number of independent SNPs in the chromosome 18 region between positions 55,850,000–56,230,000. The number of independent SNPs was determined by selecting tagSNPs from HapMap3 (release 27) CEU and YRI genotypes in the region using Tagger (tagSNP r^2^≥0.8, 45 CEU and 106 YRI tagSNPs selected).

## Results

Association analysis was performed using adiposity-related traits (BMI, plasma leptin levels, percentage total body fat mass, abdominal subcutaneous fat, and abdominal visceral fat) measured in participants from the population-based Health ABC study. The study population contained white and black participants, and all outcomes and covariates except for physical activity estimates were significantly different by race (Table 1).

**Table 1:** Health ABC participant characteristics by race.

	White		Black		
Characteristic	*n*	Mean ±SD or *n* (%)	*n*	Mean ±SD or *n* (%)	*P*<0.05
Age (y)	1655	73.79±2.86	1175	73.45±2.89	*
Sex (males)		873 (53%)		501 (43%)	*
Height (m)	1655	1.67±0.09	1175	1.65±0.09	*
Weight (kg)	1655	74.23±14.38	1175	78.26±15.65	*
BMI (kg/m^2^)	1655	26.57±4.15	1175	28.70±5.46	*
Body Fat (%)	1592	34.75±7.19	1136	35.51±8.64	*
Leptin (ng/ml)	1635	12.81±11.58	1152	17.31±13.92	*
VAT (cm^2^)	1590	152.01±69.03	1128	130.38±61.59	*
SAT (cm^2^)	1558	265.77±102.72	1074	315.60±139.75	*
Physical Activity (Kcal/kg/week)	1655	84.02±62.83	1175	79.91±75.94	
Prevalent Diabetes	-	319 (20%)	-	347 (31%)	*
Education					*
Not high school graduate	-	203 (12%)	-	507 (43%)	
High school graduate	-	564 (34%)	-	360 (31%)	
Postsecondary education	-	886 (54%)	-	303 (26%)	
Smoking habits					*
Never smoker	-	709 (43%)	-	518 (44%)	
Former smoker	-	836 (51%)	-	465 (40%)	
Current smoker	-	108 (7%)	-	189 (16%)	
Drinking habits					*
Never drinker	-	405 (25%)	-	378 (32%)	
Former drinker	-	278 (17%)	-	342 (29%)	
Current drinker	-	964 (59%)	-	450 (38%)	

VAT =  abdominal visceral adipose tissue. SAT =  abdominal subcutaneous adipose tissue.

*p<0.05 between races by t-test for continuous traits and by Chi-squared test for categorical traits.

### Replication analysis

We first attempted to replicate the previous finding that the minor alleles of two nsSNPs (rs52820871 and rs2229616) have a protective effect on obesity that is stronger in females [12], [39]. While the association between the minor alleles of these two nsSNPs and lower BMI did not reach statistical significance among Health ABC white participants (Table 2), the association did reach statistical significance among white females (rs52820871 *P*-value  = 0.03, rs2229616 *P*-value  = 0.01) (Table 3). Rs2229616 did show a significant interaction with sex (*P*-value*_INT_*  = 0.002), but rs52820871 did not. The minor alleles of the two nsSNPs were also associated with lower BMI in black participants and black females, but the association did not reach statistical significance (Table S1 and Table S2). Among black participants in sex-stratified analysis, the only significant association between either of the nsSNPs and adiposity-related traits was between the 251Leu allele of rs52820871 and higher abdominal visceral fat in black females (β±SE = 3.16±1.39, *P*-value = 0.02) (Table S1 and Table S2).

**Table 2:** SNP associations with adiposity-related traits in Health ABC white participants.

			BMI		% Body Fat		Leptin		Leptin^b^		VAT		SAT	
			(*n* = 1599–1682)		(*n* = 1538–1616)		(*n* = 1580–1663)		(*n* = 1519–1597)		(*n* = 1538–1620)		(*n* = 1506–1586)	
SNP^a^	Alleles	CAF	β±SE	*P* c	β±SE	*P* c	β±SE	*P* c	β±SE	*P* c	β±SE	*P* c	β±SE	*P* c
*rs52820871*	A/C	0.01	−1.07±0.75	NS	−0.28±0.96	NS	−0.16±0.24	NS	−0.14±0.17	NS	−0.45±0.36	NS	0.29±0.31	NS
*rs2229616*	G/A	0.02	−0.46±0.55	NS	−0.65±0.68	NS	−0.15±0.17	NS	−0.04±0.12	NS	0.09±0.26	NS	−0.14±0.22	NS
*rs17782313*	T/C	0.22	0.24±0.17	NS	0.25±0.21	NS	0.05±0.05	NS	0.01±0.04	NS	−0.22±0.08	0.006	0.03±0.07	NS
*rs11152221*	C/T	0.31	0.84±0.20	2×10^−5^ *	0.75±0.25	0.003*	0.22±0.06	4×10^−4^ *	0.07±0.05	NS	0.07±0.10	NS	0.02±0.08	NS
*rs1943225*	T/G	0.23	0.61±0.45	NS	−0.08±0.56	NS	0.29±0.14	0.04	0.29±0.10	0.004	−0.27±0.22	NS	−0.21±0.18	NS

Alleles listed as reference allele/coded allele. CAF =  coded allele frequency. VAT =  abdominal visceral adipose tissue. SAT =  abdominal subcutaneous adipose tissue.

a Additive coding  = rs52820871/I251L, rs2229616/V103I, and rs17782313; dominant coding  = rs11152221; recessive coding  = rs1943225.

b Leptin outcome adjusted for percentage of body fat.

c Unadjusted *P*-value. *P*-values >0.05 shown as NS (not significant).

*Empirical *P*-value ≤0.05.

**Table 3:** SNP associations with adiposity-related traits in Health ABC white female and white male participants.

White female participants														
			BMI		% Body Fat		Leptin		Leptin^b^		VAT		SAT	
			(*n* = 748–786)		(*n* = 722–756)		(*n* = 737–775)		(*n* = 711–745)		(*n* = 723–760)		(*n* = 707–742)	
SNP^a^	Alleles	CAF	β±SE	*P* c	β±SE	*P* c	β±SE	*P* c	β±SE	*P* c	β±SE	*P* c	β±SE	*P* c
*rs52820871*	A/C	0.01	−2.48±1.16	0.03	−1.88±1.49	NS	−0.41±0.39	NS	−0.15±0.29	NS	−0.38±0.49	NS	0.26±0.47	NS
*rs2229616*	G/A	0.02	−2.27±0.88	0.01	−1.38±1.09	NS	−0.42±0.30	NS	−0.16±0.21	NS	−0.19±0.38	NS	0.34±0.34	NS
*rs17782313*	T/C	0.22	0.36±0.26	NS	0.29±0.33	NS	0.06±0.09	NS	−0.01±0.06	NS	−0.13±0.11	NS	−0.04±0.10	NS
*rs11152221*	C/T	0.31	1.18±0.31	2×10^−4^ *	0.81±0.39	0.04	0.25±0.11	0.02	0.08±0.08	NS	0.18±0.13	NS	−0.09±0.12	NS
*rs1943225*	T/G	0.23	0.69±0.69	NS	−0.83±0.86	NS	0.45±0.23	0.05	0.61±0.17	2×10^−4^ *	−0.10±0.29	NS	−0.48±0.26	NS

White male participants														
			BMI		% Body Fat		Leptin		Leptin^b^		VAT		SAT	
			(*n* = 851–896)		(*n* = 816–860)		(*n* = 843–888)		(*n* = 808–852)		(*n* = 815–860)		(*n* = 799–844)	
SNP^a^	Alleles	CAF	β±SE	*P* c	β±SE	*P* c	β±SE	*P* c	β±SE	*P* c	β±SE	*P* c	β±SE	*P* c
*rs52820871*	A/C	0.01	0.13±0.97	NS	0.97±1.23	NS	0.09±0.27	NS	−0.04±0.20	NS	−0.54±0.53	NS	0.46±0.40	NS
*rs2229616*	G/A	0.02	1.07±0.67	NS	−0.04±0.86	NS	0.06±0.19	NS	0.08±0.14	NS	0.33±0.37	NS	−0.37±0.29	NS
*rs17782313*	T/C	0.22	0.11±0.21	NS	0.20±0.27	NS	0.04±0.06	NS	0.03±0.04	NS	−0.34±0.12	0.004	0.08±0.09	NS
*rs11152221*	C/T	0.31	0.57±0.24	0.02	0.68±0.32	0.03	0.19±0.07	0.005	0.07±0.05	NS	−0.01±0.14	NS	0.08±0.11	NS
*rs1943225*	T/G	0.23	0.76±0.57	NS	0.84±0.74	NS	0.15±0.16	NS	−0.01±0.12	NS	−0.38±0.32	NS	0.10±0.25	NS

Alleles listed as reference allele/coded allele. CAF =  coded allele frequency. VAT =  abdominal visceral adipose tissue. SAT =  abdominal subcutaneous adipose tissue.

a Additive coding  = rs52820871/I251L, rs2229616/V103I, and rs17782313; dominant coding  = rs11152221; recessive coding  = rs1943225.

b Leptin outcome adjusted for percentage of body fat.

c Unadjusted *P*-value. *P*-values >0.05 shown as NS (not significant).

*Empirical *P*-value ≤0.05.

The previously identified top hit in the distal LD block 3′ of *MC4R*, rs17782313, was associated with higher BMI in all race and sex stratified analyses, but the association failed to reach statistical significance (Table 2, Table 3, and Table S2). Our study did not have sufficient power (Power  = 0.25, 2-sided α = 0.05) to detect the reported effect size (0.22 BMI units) for the association between rs17782313 and BMI [19]. The SNP rs17782313 was significantly associated with lower abdominal visceral fat in white and white male participants (*P*-value  = 0.006 and 0.004, respectively) (Table 2 and Table 3). In addition, rs17782313 was significantly associated with higher leptin levels in black and black female participants (*P*-value  = 0.03 and 0.05, respectively) (Table S2).

### Genetic associations in the MC4R gene region

**Figure 1:**
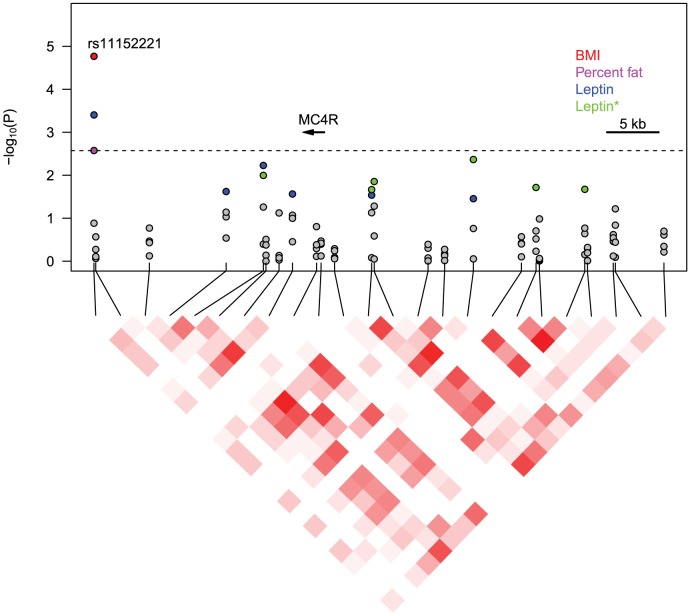
SNP associations in and near *MC4R* with adiposity in white Health ABC participants. SNP genotypes from custom Illumina Golden Gate array. Gray points indicate association *P*-value >0.05. Non-gray points indicate significant (*P*-value ≤0.05) associations with an adiposity trait of the corresponding color in the legend. Leptin* indicates association *P*-value for leptin adjusted for percent body fat. Dashed line indicates cut-off value for empirical *P*-value ≤0.05. LD heatmap indicates higher *r* ^2^ measures with darker red colors.

TagSNPs were selected to capture common genetic variation within the *MC4R* gene and the surrounding non-coding DNA. Two SNPs, rs11152221 and rs1943225, remained significantly associated with adiposity-related traits after correction for multiple testing in race stratified or race and sex stratified analysis (Figure 1, Figure S1). Rs11152221 was in high LD with the previously reported top association signal in the proximal LD block, rs17700633 (HapMap CEU *r* ^2^ = 0.79). After correction for multiple testing, rs11152221 coded with a dominant mode of inheritance was significantly associated with higher BMI (*P*-value_nom_ = 2×10^−5^, *P*-value*_emp_* = 5×10^−4^), percentage body fat (*P*-value_nom_ = 0.003, *P*-value*_emp_* = 0.05), and leptin levels (*P*-value_nom_ = 4×10^−4^, *P*-value*_emp_* = 0.008) among white participants, but not black participants (Table 2, Table S2, and Figure 1). Conditional analysis demonstrated that the association between rs11152221 and BMI was not dependent on rs17782313 or the two nsSNPs within the *MC4R* gene (rs52820871 and rs2229616) (data not shown). While rs11152221 was more significantly associated with BMI in white females (*P*-value_nom_ = 2×10^−4^, *P*-value*_emp_* = 0.004) than white males (*P*-value_nom_ = 0.02, *P*-value*_emp_*>0.05), the effect estimates were not significantly different as evidenced by the lack of significance of a sex interaction term and the overlap of the 95% confidence intervals for the estimates in these two groups (Table 3). Coded with an additive mode of inheritance, rs11152221 was significantly associated in white participants with BMI (β±SE = 0.53±0.15, *P*-value  = 5×10^−4^), percentage body fat (β±SE = 0.42±0.19, *P*-value = 0.03), and leptin levels (β±SE = 0.13±0.05, *P*-value  = 0.005), and the association with BMI passed multiple test correction.

Association between rs11152221 and obesity was tested among the 296 obesity cases and 1303 obesity controls selected from Health ABC white participants. The rs11152221 T allele was significantly associated with increased odds of obesity (Dominant coding: OR = 1.76, 95% CI = 1.34–2.30, *P*-value  = 4×10^−5^, Additive coding: OR = 1.46, 95% CI = 1.20–1.78, *P*-value = 2×10^-4^, Table S3).

The association between rs11152221 and obesity was also examined using cases selected from a cohort of severely obese patients from a UCSF study and controls selected from non-obese white participants from the Health ABC study (Table S4). Fewer covariates were included in the analysis of obesity association using cases from the UCSF severe obesity study compared to cases from the Health ABC study, resulting in fewer controls being excluded due to incomplete covariate information. The rs11152221 T allele was significantly associated with increased odds of obesity (OR = 1.28, 95% CI = 1.00–1.64, *P*-value  = 0.05, Table S5) under an additive mode of inheritance.

A SNP (rs1943225) located in the non-coding DNA 5′ to *MC4R* remained significantly associated with adiposity-related traits after correction for multiple testing (Figure S1). This SNP was significantly associated with higher leptin levels adjusted for percentage body fat in white females (*P*-value_nom_ = 2×10^−4^, *P*-value*_emp_* = 0.005) (Table 3). This association was nominally significant in white participants (*P*-value*_nom_* = 0.004), but did not remain significant after correction for multiple testing (Table 2). In white females, rs1943225 was associated with lower percentage body fat but higher plasma leptin levels (Table 3). As percentage body fat and leptin levels are strongly correlated among Health ABC white participants (Pearson's *r* = 0.77), it is expected that an association with lower body fat could mask an association with higher leptin levels. Thus, when leptin levels are adjusted for percentage body fat, the association between rs1943225 and higher leptin levels was observed to be highly significant in white females (Table 3). This association was only observed when rs1943225 was coded with a recessive mode of inheritance. When the six genotyped tagSNPs (rs1943217, rs1943218, rs8093815, rs9965495, rs17066879, and rs17773774) that were in LD with rs1943225 (r^2^>0.6) were also coded with a recessive mode of inheritance, three of them remained significantly associated after multiple test correction with leptin levels adjusted for percentage body fat in white females (rs1943217, rs1943218, and rs9965495) (Figure S1).

**Figure 2:**
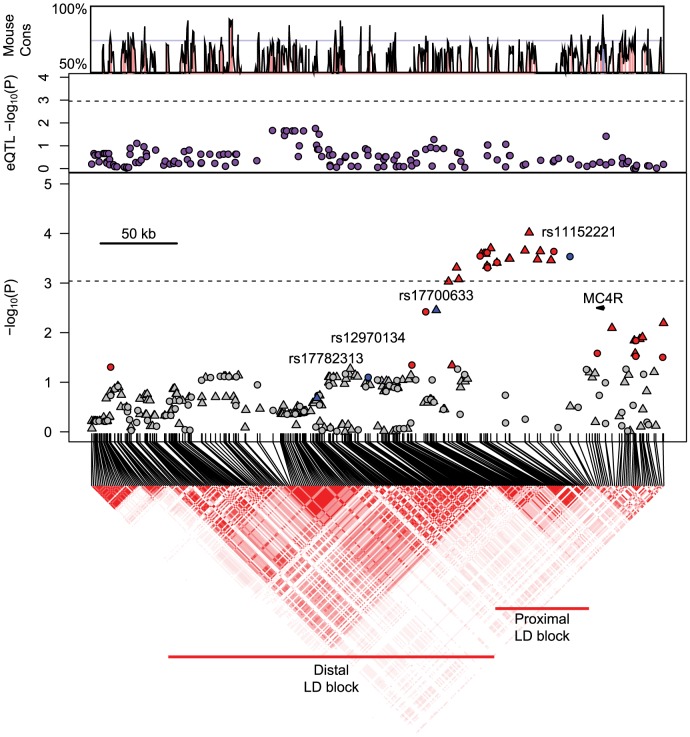
Association between BMI and genotyped and imputed SNPs in white Health ABC participants. SNP genotypes from genome-wide Illumina array. In the panel displaying BMI association *P*-values, circles mark directly genotyped SNPs and triangles mark imputed SNPs. Gray points indicate association *P*-value >0.05. Red points indicate significant (*P*-value ≤0.05) associations with BMI. Anchor SNPs colored in blue. Purple circles mark SNP association with *MC4R* expression in HapMap CEU lymphoblastoid cell lines. In the panels showing trait association and eQTL *P*-values, the dashed line indicates cut-off value for Bonferroni-corrected *P*-value ≤0.05. LD heatmap indicates higher *r* ^2^ measures with darker red colors. Nucleotide conservation between the human and mouse is indicated on the top panel of the figure and was obtained using the VISTA browser.

**Figure 3:**
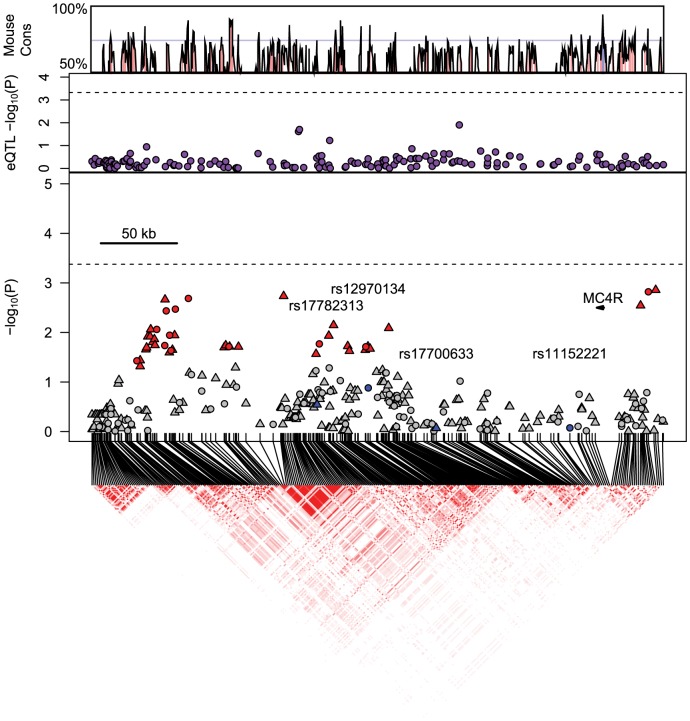
Association between BMI and genotyped and imputed SNPs in black Health ABC participants. SNP genotypes from genome-wide Illumina array. Circles mark directly genotyped SNPs and triangles mark imputed SNPs. Gray points indicate association *P*-value >0.05. Red points indicate significant (*P*-value ≤0.05) associations with BMI. Anchor SNPs colored in blue. Purple circles mark SNP association with *MC4R* expression in HapMap YRI lymphoblastoid cell lines. In the panels showing trait association and eQTL *P*-values, the dashed line indicates cut-off value for Bonferroni-corrected *P*-value ≤0.05. LD heatmap indicates higher *r* ^2^ measures with darker red colors. Nucleotide conservation between the human and mouse is indicated on the top panel of the figure and was obtained using the VISTA browser.

The location of the most significantly associated SNP, rs11152221, in the proximal 3′ LD block previously identified in GWAS meta-analyses of anthropometric traits compelled us to examine SNPs in the proximal and distal 3′ LD blocks using genome-wide genotype data in Health ABC participants. Multiple SNPs in the proximal but not the distal LD block were associated with BMI in white participants after multiple test correction (Figure 2). Two SNPs in the distal LD block (rs17782313 and rs12970134) that were previously reported to be significantly associated with BMI were not associated with BMI (*P*-value >0.05) in Health ABC white participants (Figure 2), confirming the association results found from Taqman genotyping of rs17782313 in Health ABC participants (Table 2). We next attempted to take advantage of the shorter haplotypes present in Health ABC black participants in this region, however, no SNPs were significantly associated with BMI after multiple test correction in this population (Figure 3).

### Functional characterization of non-coding regions

We investigated whether non-coding variants located 3′ of *MC4R* were associated with *MC4R* expression by using publicly available eQTL data from HapMap CEU and YRI lymphoblastoid cell lines. In the *MC4R* 288 kb gene region encompassing the proximal and distal LD blocks, no SNPs were significantly associated with *MC4R* expression after correction for multiple testing in CEU or YRI cell lines (Figure 2 and Figure 3). While lymphoblastoid cell lines are convenient for high-throughput gene expression studies, these cell lines might not accurately reflect gene expression in hypothalamic tissue. Thus, we selected a DNA region near rs11152221 in the proximal LD block to search for potential causal variants by sequencing and subsequent *in vivo* enhancer assays.

DNA regions in the human genome near rs11152221 are conserved with the mouse genome (Figure 2 and Figure 3). This SNP is 704 bp 3′ to a 357 bp stretch of DNA that is 70.6% conserved with mouse DNA and 1091 bp 3′ to a 156 bp DNA region that is 70.4% conserved. These areas of conservation, in their entirety, were sequenced in twenty severely obese patients (ten rs11152221CC homozygotes and ten rs11152221 TT homozygotes) from an ongoing UCSF study (see Materials and Methods). The twenty obese patients were all female Caucasians without diabetes, and patient characteristics did not differ by rs11152221 genotype (Table S6). Given that the rs11152221 T allele frequency was 0.31, we hypothesized that potential causal variants tagged by rs11152221 would also be common and could be detected in ten homozygous patients. However, we were unable to detect any novel homozygous variants in this region in our small sample set of severely obese patients homozygous for the rs11152221 T allele. One patient homozygous for the rs11152221 C allele (major allele) was homozygous for the minor allele of rs11872889, and one patient homozygous for the rs11152221 T allele (minor allele) was heterozygous for the minor allele of rs72973926. No association was found between rs11872889 and BMI in Health ABC white participants (n = 1613, β±SE = −0.31±0.31, *P*-value  = 0.31, coded allele (A) frequency  = 0.08, MACH r^2^ imputation quality  = 0.70). The SNP rs11872889 was not imputed in Health ABC black participants. The SNP rs72973926 was not imputed in the Health ABC cohort, and its allele frequency from the 1000 genomes project was reported for the YRI population (C allele frequency  = 0.06), but not for populations of European descent.

We next tested the conserved DNA region of interest for enhancer activity using both zebrafish and mouse enhancer assays. In zebrafish embryos examined up to 72 hours post-fertilization, the 1.25 kb conserved region amplified from a patient homozygous for the rs11152221 major allele (C) that was associated with lower values of adiposity-related traits was negative for reporter expression in the midbrain (24 hpf: midbrain expression in 1.6% of 126 examined embryos; 48 hpf: 1% of 99 embryos; 72 hpf: 0% of 87 embryos). The same DNA region amplified from the same patient homozygous for the rs11152221 C allele was also tested for enhancer activity in the mouse. At E14-15, the earliest age in which *MC4R* expression has been detected [40], all eight mouse embryos that carried the transgene (as determined by PCR) displayed minimal levels of reporter expression in the brain. The three embryos with detectable reporter expression did not display a consistent expression pattern.

**Figure 4:**
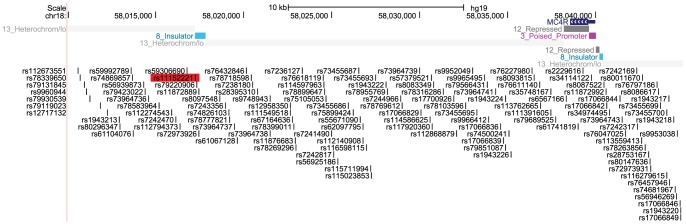
ENCODE-based transcriptional insulators near *MC4R*. Schematic depicting the genomic region (hg19 assembly) surrounding *MC4R* that contains rs11152221 (highlighted in red) and ENCODE-annotated insulators. Adapted from the UCSC Genome Browser, http://genome.ucsc.edu/ [51].

Examination of data from the Encyclopedia of DNA Elements (ENCODE) Project [26] indicated that the 1.25 kb DNA region of interest contains a possible insulator element (Figure 4). ENCODE's assignment of a chromatin state as an insulator is based on a Hidden Markov Model applied to ChIP-seq data, including ChIP-seq using an antibody against the CCCTC-binding factor (CTCF), a protein that is known to associate with insulator activity [41], [42], [43]. ENCODE data also indicated the presence of a second possible insulator element located 200 bp upstream of the *MC4R* promoter (Figure 4). These two insulators could potentially modulate interactions between enhancers and the *MC4R* promoter.

## Discussion

In this study, we examined the association between SNPs in the *MC4R* gene region with multiple measures of adiposity in a biracial study population, the Health ABC study. In addition, we used eQTL data to determine whether SNPs in the region were associated with *MC4R* expression levels, and we functionally characterized a candidate DNA region for enhancer activity *in vivo*. The associations between lower BMI and two rare non-synonymous *MC4R* SNPs replicated in white female Health ABC participants, but the association between BMI and a common SNP discovered through GWAS (rs17782313) in a distal LD block in the 3′ *MC4R* non-coding region did not. We further explored SNPs in the 3′ *MC4R* non-coding region, and discovered that SNPs in the proximal LD block, but not the distal LD block, were significantly associated with BMI after correction for multiple testing in white Health ABC participants. Within the proximal LD block, we selected a DNA region to be tested for *in vivo* enhancer activity based on the fact that it contained a SNP (rs11152221) that was significantly associated with adiposity and obesity, it was the closest DNA segment to the *MC4R* gene, and it was highly conserved with the mouse genome. However, this DNA segment failed to demonstrate enhancer activity. ENCODE data suggested that the transcriptional insulator CTCF can bind this DNA segment. Not only could the presence of a potential insulator help to explain the lack of enhancer activity in our assays, but it could also explain why non-coding *MC4R* SNPs that have been consistently associated with anthropometric and adiposity traits fail to be associated with *MC4R* RNA expression in eQTL experiments.

*Cis*-acting regulatory regions include functional elements such as enhancers and insulators [44]. Independent of their orientation and distance from the promoter, enhancers can regulate transcription and are often composed of clusters of transcription factor binding sites [44]. Neither *MC4R* transcriptional regulatory regions nor transcription factors regulating *MC4R* have been identified. Recent GWAS of adiposity-related traits have consistently identified highly significant SNP associations within two large LD blocks downstream of *MC4R*, highlighting the importance of this non-coding region, but molecular mechanisms for these SNP associations have yet to be identified [3], [18], [19], [20], [21], [22], [23]. SNPs in these non-coding regions could be in high LD with causal variants disrupting functional *MC4R* regulatory elements, but our analysis of eQTL data from HapMap lymphoblastoid cell lines failed to support this hypothesis. It is worth noting that gene expression regulation in lymphoblastoid cell lines is unlikely to accurately reflect what occurs in hypothalamic neurons, which are the relevant cell type. Thus, we took an *in vivo* enhancer assay approach using the mouse and zebrafish model systems to determine whether DNA surrounding SNPs significantly associated with adiposity can act as enhancers. While the DNA region that we examined did not act as an enhancer in our assays, ENCODE data indicated that the DNA region can bind CTCF. The associated SNP rs11152221 does not overlap with the ENCODE-predicted CTCF binding region and does not directly interrupt a CTCF binding site. Nevertheless, three potential CTCF binding sites are located within 250 bp of rs11152221, supporting CTCF binding to this DNA region (Table S7, Figure S2).

While further work will be needed to experimentally determine whether the DNA region surrounding rs11152221 does in fact bind CTCF, the ENCODE annotation and presence of potential CTCF binding sites lead to various models and testable hypotheses. One possible model invokes CTCF's role as a transcriptional insulator. CTCF binding could create a transcriptional insulator that blocks enhancers from activating the *MC4R* promoter, and genetic variation at the *MC4R* locus could modify the efficiency of CTCF binding in the region. In addition to acting as an insulator, CTCF has also been shown to play a role in transcriptional activation by forming active chromatin hubs through intra-chromosomal interactions [41]. In addition to the downstream *MC4R* DNA region that includes rs11152221 and an ENCODE-predicted CTCF-based insulator, ENCODE also predicts a CTCF-based insulator approximately 200 bp upstream of the *MC4R* transcription start site. Intra-chromosomal interactions between these two potential CTCF binding sites could bring the DNA region spanning the two large LD blocks, which contain SNPs that are significantly associated with adiposity-related traits, in close proximity to the *MC4R* promoter. CTCF has been shown to regulate interactions between promoters and distant enhancers by forming chromosomal loops. The developmental timing of the expression of genes at the *β-globin* locus (ε, Gγ, Aγ, δ, and β) is regulated by CTCF-mediated intra-chromosomal looping between the locus control region (LCR) and the promoter of the gene to be expressed [45]. At the *CFTR* gene, CTCF binds downstream of the gene and interacts with the *CFTR* promoter through a chromosomal loop, which is proposed to create an active chromatin hub [46]. Similar to CTCF's role at these loci, CTCF could potentially facilitate *MC4R* expression through chromosomal loop formation.

A previous study conducted using participants in the Health ABC study and the Age Gene/Environment Susceptibility-Reykjavik (AGES-Reykjavik) study examined whether reported BMI-associated SNPs were associated with anthropometric and adiposity-related traits in the elderly [47]. The single SNP they examined in the *MC4R* region, rs571312, is located in the distal LD block, and not surprisingly, no association with BMI was identified. We also found no evidence for association between SNPs in the distal LD block and BMI, but by examining the entire genomic region, we identified highly significant SNP associations with BMI in the proximal LD block, thus highlighting the importance of the examination of the entire *MC4R* gene region.

Despite the significant SNP associations in non-coding DNA downstream of *MC4R* that we observed in Health ABC white participants, we did not observe significant SNP associations in these DNA regions in Health ABC black participants. There were fewer black participants in the Health ABC study than white participants, resulting in a loss of power in the analysis of SNP associations in black participants. A previously reported GWAS of BMI performed in individuals of African ancestry failed to identify SNP associations reaching genome-wide significance levels, but nominally significant SNP associations were identified near the 3′ distal LD block of *MC4R* [48]. Previously reported BMI-associated SNPs from populations of European descent were evaluated in a meta-analysis of SNP associations with BMI in six cohorts composed of individuals of African ancestry (n = 4992), and 2 of the 7 SNPs examined at the *MC4R* locus were nominally significant (*P*-value ≤0.05) [49]. A GWAS meta-analysis of BMI performed in a total of 71,412 individuals of African ancestry, in which Health ABC black participants contributed to 1.6% of the sample size, identified a genome-wide significant SNP association (rs6567160) near the distal LD block downstream of *MC4R* [50]. At the *MC4R* locus, the most significant SNP in African Americans (rs6567160) was not in LD (AFR r^2^ = 0.03) with the most significant SNP reported in individuals of European ancestry (rs571312), and rs571312 was not nominally associated with BMI in African Americans [50]. Taken together, these results indicate that SNPs downstream of *MC4R* are significantly associated with BMI in African Americans, but allelic heterogeneity is likely to exist.

In addition to the low power for our analysis of SNP associations in black participants from the Health ABC study, there were also limitations to our case-control study using cases from the UCSF study of severe obesity. Specifically, the cases were younger and the percentage of females was higher compared to controls. Regression models adjusted for the effect of sex. However, the nearly perfect case-control separation by age prevented the assessment of the confounding effect of age. Only a single case (aged 70 years) overlapped with the age range of controls (minimum age of controls 69 years).

In summary, the DNA region downstream of *MC4R* containing our most significantly associated SNP did not act as an enhancer, but genomic annotation by ENCODE led us to a proposed model where intra-chromosomal interactions mediated by CTCF could bring a region containing SNPs significantly associated with adiposity in close proximity to the *MC4R* promoter. Our study draws attention to the region of the proximal LD block containing this putative insulator. This information could help to guide studies aimed at identifying the molecular mechanisms of genetic associations with adiposity in the *MC4R* region.

## Supporting Information

Figure S1 **SNP associations in and near** ***MC4R*** **with adiposity in white female Health ABC participants.** SNP genotypes from custom Illumina Golden Gate array. Gray points indicate association *P*-value >0.05. Non-gray points indicate significant (*P*-value ≤0.05) associations with an adiposity trait of the corresponding color in the legend. Dashed line indicates cut-off value for empirical *P*-value ≤0.05. LD heatmap indicates higher *r* ^2^ measures with darker red colors.
(EPS)

Figure S2 **CTCF position weight matrix from JASPAR core.**
(EPS)

Table S1 **Adiposity-related traits in the Health ABC cohort by SNP genotype and race.**
(DOCX)

Table S2 **SNP associations with adiposity-related traits in Health ABC black participants and stratified by sex.**
(DOCX)

Table S3 **Obesity association with rs11152221 using cases and controls from Health ABC white participants.**
(DOCX)

Table S4 **Characteristics of participants in case-control obesity study with cases from UCSF study.**
(DOCX)

Table S5 **Obesity association with rs11152221 using cases from UCSF study.**
(DOCX)

Table S6 **Characteristics of sequenced patients from UCSF study.**
(DOCX)

Table S7 **CTCF binding sites within 250 bp of rs11152221.**
(DOCX)

Text S1 **ARRIVE guidelines checklist.**
(DOC)
