# Supplementary material for: Genetic Association Study of Adiposity and Melanocortin-4 Receptor (MC4R) Common Variants: Replication and Functional Characterization of Non-Coding Regions
Source: PLoS One. 2014 May 12;9(5):e96805. doi: 10.1371/journal.pone.0096805 (PMC4018404; doi:10.1371/journal.pone.0096805)
Supplement: Table S1 — Adiposity-related traits in the Health ABC cohort by SNP genotype and race. (DOCX) [file pone.0096805.s003.docx]

**Table S1.** Adiposity-related traits in the Health ABC cohort by SNP genotype and race.

|  | *I251L/rs52820871* | | | | | | | | | | | | | | | | | | | | | | | | | | | | | |
| --- | --- | --- | --- | --- | --- | --- | --- | --- | --- | --- | --- | --- | --- | --- | --- | --- | --- | --- | --- | --- | --- | --- | --- | --- | --- | --- | --- | --- | --- | --- |
|  | Whites | | | | | | | | | | | | | | Blacks | | | | | | | | | | | | | | | |
|  | *Ile/Ile* | | | | | *Ile/Leu* | | | | | *Leu/Leu* | | | | *Ile/Ile* | | | | | *Ile/Leu* | | | | | | | *Leu/Leu* | | | |
| *Traits* | *n* | | | Mean ± SD | | *n* | Mean ± SD | | | | *n* | | | | *n* | | Mean ± SD | | | *n* | | | | Mean ± SD | | | *n* | | | |
| BMI (kg/m^2^) | 1625 | | | 26.6 ± 4.1 | | 30 | 25.4 ± 4.2 | | | | 0 | | | | 1169 | | 28.7 ± 5.5 | | | 5 | | | | 25.8 ± 3.6 | | | 0 | | | |
| % body fat | 1563 | | | 34.8 ± 7.2 | | 29 | 34.4 ± 6.8 | | | | 0 | | | | 1130 | | 35.5 ± 8.6 | | | 5 | | | | 31.7 ± 12.1 | | | 0 | | | |
| Leptin (ng/ml) | 1605 | | | 12.8 ± 11.6 | | 30 | 11.6 ± 12.1 | | | | 0 | | | | 1146 | | 17.3 ± 13.9 | | | 5 | | | | 12.1 ± 9.7 | | | 0 | | | |
| VAT (cm^2^) | 1560 | | | 152.4 ± 68.9 | | 30 | 131 ± 72.2 | | | | 0 | | | | 1122 | | 130.4 ± 61.6 | | | 5 | | | | 131.9 ± 64.1 | | | 0 | | | |
| SAT (cm^2^) | 1530 | | | 266.0 ± 102.8 | | 28 | 254.9 ± 98.5 | | | | 0 | | | | 1068 | | 316.1 ± 139.8 | | | 5 | | | | 238.0 ± 131.6 | | | 0 | | | |
|  | *V103I/rs2229616* | | | | | | | | | | | | | | | | | | | | | | | | | | | | | |
|  | Whites | | | | | | | | | | | | | | Blacks | | | | | | | | | | | | | | | |
|  | *Val/Val* | | | | *Val/Ile* | | | | | *Ile/Ile* | | | | | *Val/Val* | | | | | | *Val/Ile* | | | | | | *Ile/Ile* | | | |
| *Traits* | *n* | Mean ± SD | | | *n* | | | | Mean ± SD | *n* | | | | Value | *n* | | | Mean ± SD | | | *n* | | | | Mean ± SD | | *n* | | | |
| BMI (kg/m^2^) | 1601 | 26.6 ± 4.1 | | | 52 | | | | 25.8 ± 4.2 | 1 | | | | 26.3 | 1128 | | 28.7 ± 5.5 | | | | | 47 | | 28.2 ± 4.7 | | | 0 | | | |
| % body fat | 1538 | 34.8 ± 7.2 | | | 52 | | | | 34.6 ± 7.1 | 1 | | | | 24.9 | 1089 | | 35.5 ± 8.6 | | | | | 47 | | 34.8 ± 9.1 | | | 0 | | | |
| Leptin (ng/ml) | 1581 | 12.8 ± 11.7 | | | 52 | | | | 11.8 ± 8.7 | 1 | | | | 2.6 | 1105 | | 17.3 ± 13.9 | | | | | 47 | | 17.4 ± 13.6 | | | 0 | | | |
| VAT (cm^2^) | 1537 | 152.1 ± 69.1 | | | 51 | | | | 148.9 ± 67.8 | 1 | | | | 195.3 | 1083 | | 130.9 ± 61.9 | | | | | 45 | | 118.5 ± 52.8 | | | 0 | | | |
| SAT (cm^2^) | 1508 | 266.1 ± 103.3 | | | 48 | | | | 260.4 ± 83.1 | 1 | | | | 162.3 | 1030 | | 315.3 ± 139.4 | | | | | 44 | | 323.0 ± 149.9 | | | 0 | | | |
|  | *rs17782313* | | | | | | | | | | | | | | | | | | | | | | | | | | | | | |
|  | Whites | | | | | | | | | | | | | | Blacks | | | | | | | | | | | | | | | |
|  | *T/T* | | | | *T/C* | | | | | *C/C* | | | | | *T/T* | | | | | | | *T/C* | | | | | *C/C* | | | |
| *Traits* | *n* | Mean ± SD | | | *n* | | | | Mean ± SD | *n* | | | | Mean ± SD | *n* | | | | Mean ± SD | | | *n* | | | Mean ± SD | | *n* | | Mean ± SD | |
| BMI (kg/m^2^) | 1035 | 26.4 ± 4.1 | | | 620 | | | | 26.8 ± 4.2 | 77 | | | | 26.7 ± 4.1 | 633 | | | | 28.6 ± 5.5 | | | 481 | | 28.6 ± 5.3 | | | 92 | | 29.6 ± 5.8 | |
| % body fat | 990 | 34.4 ± 7.0 | | | 602 | | | | 35.0 ± 7.5 | 72 | | | | 35.4 ± 6.8 | 618 | | | | 35.3 ± 8.7 | | | 460 | | 35.3 ± 8.6 | | | 90 | | 36.9 ± 8.4 | |
| Leptin (ng/ml) | 1020 | 12.3 ± 11.0 | | | 615 | | | | 13.4 ± 12.1 | 77 | | | | 13.2 ± 12.5 | 621 | | | | 16.8 ± 13.6 | | | 473 | | 17.2 ± 14.4 | | | 89 | | 19.7 ± 12.6 | |
| VAT (cm^2^) | 1001 | 153.4 ± 70.0 | | | 590 | | | | 152.1 ± 69.5 | 75 | | | | 143.8 ± 66.0 | 606 | | | | 128.5 ± 60.7 | | | 460 | | 128.8 ± 62.6 | | | 92 | | 142.5 ± 62.5 | |
| SAT (cm^2^) | 983 | 260.1 ± 99.3 | | | 578 | | | | 273.2 ± 107.5 | 71 | | | | 268.8 ± 95.4 | 577 | | | | 313.2 ± 141.8 | | | 440 | | 314.4 ± 137.9 | | | 87 | | 331.4 ± 133.8 | |
|  | *rs11152221* | | | | | | | | | | | | | | | | | | | | | | | | | | | | | |
|  | Whites | | | | | | | | | | | | | | | Blacks | | | | | | | | | | | | | | |
|  | *C/C* | | | | *C/T* | | | | | | | *T/T* | | | | *C/C* | | | | | | *C/T* | | | | | | *T/T* | | |
| *Traits* | *n* | | | Mean ± SD | *n* | | | Mean ± SD | | | | *n* | Mean ± SD | | | *n* | | | Mean ± SD | | | *n* | | | | Mean ± SD | | *n* | | Mean ± SD |
| BMI (kg/m^2^) | 781 | | | 26.1 ± 3.8 | 722 | | | 27.0 ± 4.4 | | | | 147 | 26.8 ± 4.0 | | | 241 | | | 28.4 ± 5.1 | | | 555 | | | | 28.8 ± 5.5 | | 369 | | 28.7 ± 5.6 |
| % body fat | 754 | | | 34.2 ± 7.1 | 690 | | | 35.3 ± 7.3 | | | | 143 | 35.0 ± 7.3 | | | 231 | | | 35.1 ± 8.7 | | | 534 | | | | 35.7 ± 8.6 | | 361 | | 35.5 ± 8.7 |
| Leptin (ng/ml) | 770 | | | 11.8 ± 10.7 | 714 | | | 13.8 ± 12.4 | | | | 146 | 13.0 ± 11.3 | | | 237 | | | 17.3 ± 14.8 | | | 547 | | | | 17.5 ± 14.0 | | 358 | | 16.9 ± 13.2 |
| VAT (cm^2^) | 752 | | | 146.3 ± 67.2 | 693 | | | 158.1 ± 70.6 | | | | 140 | 148.7 ± 65.0 | | | 233 | | | 130.0 ± 63.4 | | | 532 | | | | 131.7 ± 61.6 | | 353 | | 128.7 ± 60.5 |
| SAT (cm^2^) | 734 | | | 254.9 ± 96.2 | 683 | | | 276.5 ± 107.4 | | | | 136 | 268.5 ± 104.5 | | | 229 | | | 309.4 ± 139.5 | | | 501 | | | | 320.4 ± 140.0 | | 334 | | 312.0 ± 138.9 |
|  | *rs1943225* | | | | | | | | | | | | | | | | | | | | | | | | | | | | | |
|  | Whites | | | | | | | | | | | | | | | Blacks | | | | | | | | | | | | | | |
|  | *T/T* | | | | *T/G* | | | | | | | *G/G* | | | | *T/T* | | | | | | | *T/G* | | | | | *G/G* | | |
| *Traits* | *n* | | Mean ± SD | | *n* | | | | Mean ± SD | | | *n* | Mean ± SD | | | *n* | | | Mean ± SD | | | | *n* | | | Mean ± SD | | *n* | | Mean ± SD |
| BMI (kg/m^2^) | 995 | | 26.4 ± 4.1 | | 573 | | | 26.8 ± 4.1 | | | | 86 | 27.0 ± 4.3 | | | 1020 | | | 28.6 ± 5.4 | | | | 147 | | | 29.2 ± 5.9 | | 7 | | 29.1 ± 4.7 |
| % body fat | 958 | | 34.4 ± 7.2 | | 550 | | | 35.2 ± 7.2 | | | | 84 | 35.2 ± 6.5 | | | 985 | | | 35.4 ± 8.6 | | | | 144 | | | 36.2 ± 9.2 | | 6 | | 36.9 ± 9.6 |
| Leptin (ng/ml) | 980 | | 12.6 ± 11.5 | | 569 | | | 12.8 ± 11.3 | | | | 85 | 15.6 ± 14.0 | | | 1001 | | | 17.2 ± 13.9 | | | | 143 | | | 17.9 ± 14.1 | | 7 | | 13.1 ± 10.0 |
| VAT (cm^2^) | 951 | | 151.2 ± 70.4 | | 554 | | | 153.1 ± 67.3 | | | | 84 | 153.5 ± 65.4 | | | 982 | | | 129.8 ± 60.9 | | | | 138 | | | 134.6 ± 66.9 | | 7 | | 122.8 ± 51.4 |
| SAT (cm^2^) | 935 | | 261.6 ± 101.7 | | 541 | | | 272.2 ± 105.5 | | | | 81 | 270.9 ± 94.5 | | | 933 | | | 313.9 ± 139.0 | | | | 134 | | | 322.8 ± 144.4 | | 6 | | 418.8 ± 150.6 |

None of the trait values are transformed or adjusted for covariates. VAT = abdominal visceral adipose tissue. SAT = abdominal subcutaneous adipose tissue.
