# Supplementary material for: Genetic Association Study of Adiposity and Melanocortin-4 Receptor (MC4R) Common Variants: Replication and Functional Characterization of Non-Coding Regions
Source: PLoS One. 2014 May 12;9(5):e96805. doi: 10.1371/journal.pone.0096805 (PMC4018404; doi:10.1371/journal.pone.0096805)
Supplement: Table S2 — SNP associations with adiposity-related traits in Health ABC black participants and stratified by sex. (DOCX) [file pone.0096805.s004.docx]

**Table S2.** SNP associations with adiposity-related traits in Health ABC black participants and stratified by sex.

| Black participants | | | | | | | | | | | | | | | | | | | | | | |
| --- | --- | --- | --- | --- | --- | --- | --- | --- | --- | --- | --- | --- | --- | --- | --- | --- | --- | --- | --- | --- | --- | --- |
|  |  | |  | BMI | | % Body Fat | | Leptin | | | | Leptin ^b^ | | | | VAT | | | | SAT | | |
|  |  | |  | (*n* = 1106 - 1145) | | (*n* = 1070 - 1110) | | (*n* = 1084 - 1123) | | | | (*n* = 1049 - 1089) | | | | (*n* = 1062 - 1100) | | | | (*n* = 1506 - 1586) | | |
| SNP ^a^ | Alleles | | CAF | β ± SE | *P* ^c^ | β ± SE | *P* ^c^ | β ± SE | | *P* ^c^ | | β ± SE | | *P* ^c^ | | β ± SE | | *P* ^c^ | | β ± SE | | *P* ^c^ |
| *rs52820871* | | A/C | 0.002 | -2.52 ± 2.27 | NS | -2.09 ± 2.50 | NS | -0.17 ± 0.58 | | NS | | 0.16 ± 0.43 | | NS | | 1.17 ± 0.92 | | NS | | -0.33 ± 0.77 | | NS |
| *rs2229616* | | G/A | 0.02 | -0.61 ± 0.76 | NS | -0.84 ± 0.84 | NS | 0.05 ± 0.19 | | NS | | 0.16 ± 0.14 | | NS | | -0.35 ± 0.31 | | NS | | 0.31 ± 0.27 | | NS |
| *rs17782313* | | T/C | 0.28 | 0.25 ± 0.24 | NS | 0.47 ± 0.26 | NS | 0.13 ± 0.06 | | 0.03 | | 0.05 ± 0.04 | | NS | | 0.07 ± 0.10 | | NS | | 0.10 ± 0.08 | | NS |
| *rs11152221* | | C/T | 0.55 | -0.15 ± 0.33 | NS | -0.02 ± 0.37 | NS | -0.08 ± 0.09 | | NS | | -0.06 ± 0.06 | | NS | | -0.10 ± 0.14 | | NS | | 0.02 ± 0.12 | | NS |
| *rs1943225* | | T/G | 0.07 | 0.20 ± 1.92 | NS | 0.83 ± 2.28 | NS | -0.71 ± 0.49 | | NS | | -0.54 ± 0.39 | | NS | | -0.45 ± 0.78 | | NS | | 0.92 ± 0.71 | | NS |
| Black female participants | | | | | | | | | | | | | | | | | | | | | | |
|  |  | |  | BMI | | % Body Fat | | Leptin | | | Leptin ^b^ | | | | VAT | | | | SAT | | | |
|  |  | |  | (*n* = 635 - 654) | | (*n* = 612 - 632) | | (*n* = 615 - 634) | | | (*n* = 593 - 613) | | | | (*n* = 609 - 627) | | | | (*n* = 577 - 595) | | | |
| SNP ^a^ | Alleles | | CAF | β ± SE | *P* ^c^ | β ± SE | *P* ^c^ | β ± SE | *P* ^c^ | | β ± SE | | *P* ^c^ | | β ± SE | | *P* ^c^ | | β ± SE | | *P* ^c^ | |
| *rs52820871* | | A/C | 0.002 | -1.84 ± 3.98 | NS | 2.16 ± 4.14 | NS | -0.31 ± 1.00 | NS | | -0.63 ± 0.75 | | NS | | 3.16 ± 1.39 | | 0.02 | | 0.70 ± 1.23 | | NS | |
| *rs2229616* | | G/A | 0.02 | -0.26 ± 1.21 | NS | -0.18 ± 1.17 | NS | 0.19 ± 0.28 | NS | | 0.17 ± 0.21 | | NS | | -0.08 ± 0.40 | | NS | | 0.20 ± 0.36 | | NS | |
| *rs17782313* | | T/C | 0.28 | 0.24 ± 0.34 | NS | 0.33 ± 0.36 | NS | 0.17 ± 0.09 | 0.05 | | 0.10 ± 0.07 | | NS | | -0.04 ± 0.12 | | NS | | 0.09 ± 0.11 | | NS | |
| *rs11152221* | | C/T | 0.55 | 0.01 ± 0.48 | NS | 0.15 ± 0.51 | NS | -0.13 ± 0.12 | NS | | -0.10 ± 0.09 | | NS | | -0.01 ± 0.17 | | NS | | -0.07 ± 0.16 | | NS | |
| *rs1943225* | | T/G | 0.07 | 0.71 ± 2.53 | NS | 2.94 ± 2.94 | NS | -0.47 ± 0.64 | NS | | -0.45 ± 0.53 | | NS | | -0.43 ± 0.90 | | NS | | 0.94 ± 0.79 | | NS | |
| Black male participants | | | | | | | | | | | | | | | | | | | | | | |
|  |  | |  | BMI | | % Body Fat | | Leptin | | | Leptin ^b^ | | | | VAT | | | | SAT | | | |
|  |  | |  | (*n* = 471 - 491) | | (*n* = 458 - 478) | | (*n* = 469 - 489) | | | (*n* = 593 - 613) | | | | (*n* = 453 - 473) | | | | (*n* = 434 - 454) | | | |
| SNP ^a^ | Alleles | | CAF | β ± SE | *P* ^c^ | β ± SE | *P* ^c^ | β ± SE | *P* ^c^ | | β ± SE | | *P* ^c^ | | β ± SE | | *P* ^c^ | | β ± SE | | *P* ^c^ | |
| *rs52820871* | | A/C | 0.002 | -2.48 ± 2.48 | NS | -4.87 ± 3.01 | NS | -0.27 ± 0.65 | NS | | 0.50 ± 0.45 | | NS | | 0.10 ± 1.22 | | NS | | -1.13 ± 1.00 | | NS | |
| *rs2229616* | | G/A | 0.02 | -0.91 ± 0.98 | NS | -1.44 ± 1.19 | NS | -0.09 ± 0.26 | NS | | 0.13 ± 0.18 | | NS | | -0.74 ± 0.49 | | NS | | 0.36 ± 0.41 | | NS | |
| *rs17782313* | | T/C | 0.28 | 0.33 ± 0.31 | NS | 0.71 ± 0.38 | NS | 0.06 ± 0.08 | 0.05 | | -0.05 ± 0.06 | | NS | | 0.22 ± 0.16 | | NS | | 0.11 ± 0.13 | | NS | |
| *rs11152221* | | C/T | 0.55 | -0.31 ± 0.43 | NS | -0.22 ± 0.52 | NS | -0.06 ± 0.11 | NS | | -0.04 ± 0.08 | | NS | | -0.20 ± 0.21 | | NS | | 0.11 ± 0.18 | | NS | |
| *rs1943225* | | T/G | 0.07 | 0.41 ± 3.03 | NS | -1.96 ± 3.69 | NS | -0.78 ± 0.79 | NS | | -0.43 ± 0.55 | | NS | | -0.91 ± 1.49 | | NS | | 0.70 ± 1.75 | | NS | |

Alleles listed as reference allele/coded allele. CAF = coded allele frequency. VAT = abdominal visceral adipose tissue. SAT = abdominal subcutaneous adipose tissue.

^a^ Additive coding = rs52820871/ I251L, rs2229616/ V103I, and rs17782313; dominant coding = rs11152221; recessive coding = rs1943225.

^b^ Leptin outcome adjusted for percentage of body fat.

^c^ Unadjusted *P*-value. *P*-values > 0.05 shown as NS (not significant).
