# Supplementary material for: Genetic Association Study of Adiposity and Melanocortin-4 Receptor (MC4R) Common Variants: Replication and Functional Characterization of Non-Coding Regions
Source: PLoS One. 2014 May 12;9(5):e96805. doi: 10.1371/journal.pone.0096805 (PMC4018404; doi:10.1371/journal.pone.0096805)
Supplement: Table S3 — Obesity association with rs11152221 using cases and controls from Health ABC white participants. (DOCX) [file pone.0096805.s005.docx]

**Table S3.** Obesity association with rs11152221 using cases and controls from Health ABC white participants.

| SNP | Alleles ^a^ | Mode ^b^ | OR | 95% CI | *P*-value |
| --- | --- | --- | --- | --- | --- |
| rs11152221 | C/T | Additive | 1.46 | 1.20 - 1.78 | 2x10^-4^ |
|  |  | Dominant | 1.76 | 1.34 – 2.30 | 4x10^-5^ |

^a^ Reference allele/coded allele

^b^ Mode of inheritance
