# Supplementary material for: Genetic Association Study of Adiposity and Melanocortin-4 Receptor (MC4R) Common Variants: Replication and Functional Characterization of Non-Coding Regions
Source: PLoS One. 2014 May 12;9(5):e96805. doi: 10.1371/journal.pone.0096805 (PMC4018404; doi:10.1371/journal.pone.0096805)
Supplement: Table S4 — Characteristics of participants in case-control obesity study with cases from UCSF study. (DOCX) [file pone.0096805.s006.docx]

**Table S4.** Characteristics of participants in case-control obesity study with cases from UCSF study.

| Trait (unit) | Obese cases | | Non-obese controls | |
| --- | --- | --- | --- | --- |
|  | *n* | Mean ± SD or *n* (%) | *n* | Mean ± SD or *n* (%) |
| age (years) | 165 | 44.51 ± 11.23 | 1348 | 73.86 ± 2.86 |
| BMI (kg/m2) | 165 | 49.38 ± 9.69 | 1348 | 25.12 ± 2.85 |
| sex (% female) | - | 129 (78%) | - | 647 (48%) |
| rs11152221 CC | - | 69 (42%) | - | 673 (50%) |
| rs11152221 CT | - | 77 (47%) | - | 560 (42%) |
| rs11152221 TT | - | 19 (11%) | - | 115 (8%) |
