# Supplementary material for: Genetic Association Study of Adiposity and Melanocortin-4 Receptor (MC4R) Common Variants: Replication and Functional Characterization of Non-Coding Regions
Source: PLoS One. 2014 May 12;9(5):e96805. doi: 10.1371/journal.pone.0096805 (PMC4018404; doi:10.1371/journal.pone.0096805)
Supplement: Table S5 — Obesity association with rs11152221 using cases from UCSF study. (DOCX) [file pone.0096805.s007.docx]

**Table S5.** Obesity association with rs11152221 using cases from UCSF study.

| SNP | Alleles ^a^ | CAF cases ^b^ | CAF controls ^b^ | Mode ^c^ | OR | 95% CI | *P*-value |
| --- | --- | --- | --- | --- | --- | --- | --- |
| rs11152221 | C/T | 0.35 | 0.29 | Additive | 1.28 | 1.00 - 1.64 | 0.05 |
|  |  |  |  | Dominant | 1.37 | 0.98 - 1.91 | 0.06 |

^a^ Reference allele/coded allele

^b^ CAF = coded allele frequency

^c^ Mode of inheritance
