# Supplementary material for: Genetic Association Study of Adiposity and Melanocortin-4 Receptor (MC4R) Common Variants: Replication and Functional Characterization of Non-Coding Regions
Source: PLoS One. 2014 May 12;9(5):e96805. doi: 10.1371/journal.pone.0096805 (PMC4018404; doi:10.1371/journal.pone.0096805)
Supplement: Table S6 — Characteristics of sequenced patients from UCSF study. (DOCX) [file pone.0096805.s008.docx]

**Table S6.** Characteristics of sequenced patients from UCSF study.

|  | rs11152221 CC | | rs11152221 TT | |
| --- | --- | --- | --- | --- |
| Trait (unit) | (n) mean ± SD or % | range | (n) mean ± SD or % | range |
| age (years) | (10) 43.90 ± 7.45 | (31 - 57) | (10) 47.30 ± 7.33 | (33 - 58) |
| sex (% female) | (10) 100% | - | (10) 100% | - |
| BMI (kg/m2) | (10) 49.27 ± 5.42 | (42.79 - 58.49) | (10) 52.75 ± 12.31 | (38.62 - 80.90) |
