# Supplementary material for: Genetic Association Study of Adiposity and Melanocortin-4 Receptor (MC4R) Common Variants: Replication and Functional Characterization of Non-Coding Regions
Source: PLoS One. 2014 May 12;9(5):e96805. doi: 10.1371/journal.pone.0096805 (PMC4018404; doi:10.1371/journal.pone.0096805)
Supplement: Table S7 — CTCF binding sites within 250 bp of rs11152221. (DOCX) [file pone.0096805.s009.docx]

**Table S7.** CTCF binding sites within 250 bp of rs11152221.

| Start ^a^ | End ^a^ | Sequence ^b^ | Score ^c^ | Strand |
| --- | --- | --- | --- | --- |
| 58017171 | 58017189 | CATCCAGAAGGCTGCGGCT | 73% | + |
| 58017468 | 58017486 | TAGCAGGCAGCAGGCAGTA | 72% | + |
| 58017182 | 58017200 | ACCCCAGCAGCAGCCGCAG | 71% | - |

^a^ Hg19 build coordinates of sequence match. Rs11152221 hg19 position 58017249.

^b^ Matched sequence from DNA region 250 bp upstream and downstream of rs11152221. DNA region is chr 18, 58,016,999 – 58,017,499 (hg19 build).

^c^ CTCF Position Weight Matrix (PWM) similarity score/maximum CTCF PWM score. CTCF JASPAR core PWM was used and is shown in Figure S2.
